# Assessment of Oral Health Knowledge and Practices and Its Association With Sociodemographic Factors Among Government Employes and Their Clients in Kuwait: A Cross-Sectional Study

**DOI:** 10.1155/ijod/8880948

**Published:** 2025-03-27

**Authors:** Huda Nazar, Maddi Shyama, Jitendra Ariga, Sabiha Almutawa, Ozayr Mahomed

**Affiliations:** ^1^Research and Survey Division, Dental Administration, Ministry of Health, Kuwait City, Kuwait; ^2^Dental Administration, Ministry of Health, Kuwait City, Kuwait; ^3^Dasman Diabetes Institute, Kuwait City, Kuwait; ^4^Discipline of Public Health Medicine, University of KwaZulu Natal, Durban, South Africa

**Keywords:** dental flossing, fast-food consumption, knowledge, oral health, practices, smoking

## Abstract

**Background and Aim:** Oral health knowledge is a vital requirement for health-related practice. Adequate knowledge of oral health is important due to its influence to better oral health and in developing healthy oral health practices. This study aims to assess the knowledge, practices towards oral health, and its association with sociodemographic factors amongst government employes and their clients in Kuwait, as well as to provide information that could form the basis for targeted oral health promotion programs. This research also aimed at determining the pattern of oral hygiene practices of the adult employes.

**Materials and Methods:** In this cross-sectional study, data were collected at the Ministries Complex and Housing Authority by a team of trained dentists using a customized questionnaire that was translated into Arabic language. The questionnaire included questions on demographic characteristics; questions about oral health practices; nutrition; knowledge of dental and oral health; and risk factors such as smoking and water pipe use.

**Results:** Respondents in Kuwait demonstrate good oral health knowledge; however, daily dental flossing is inadequately practiced. Nutritional practices such as fast-food consumption and sugary sweet consumption are common amongst the employed adults. Males and smokers have poorer knowledge and inadequate oral hygiene practices. In multivariate analysis, employes having a post school education were significantly associated with an above median knowledge (AOR: 2.34). Male participants (AOR: 0.41) and smokers (AOR: 0.59) were significantly less likely to brush their teeth. Participants who used a water pipe (AOR: 0.57) were significantly less likely to floss their teeth.

**Conclusions:** Majority of participants had a good knowledge level towards oral health. This study highlights the need for educational interventions focusing on comprehensive oral hygiene practices among adults. Further efforts are essential to increase awareness in workplaces to help encourage adult employes in Kuwait in developing healthy oral health practices.

## 1. Introduction

An estimated 3.5 billion people or almost 45% of the world's population were affected by oral diseases in 2019 [1]. In terms of magnitude, the combined estimated number of cases of oral diseases globally was about 1 billion higher than cases of all five main noncommunicable diseases (mental disorders, cardiovascular disease, diabetes mellitus, chronic respiratory diseases, and cancers) combined [1]. The major impact of burden of oral diseases in terms of number of cases and disability adjusted life years is in lower middle income countries with Southeast Asia Region and Western Pacific Region being ranked as the highest [1]. From a financial perspective, about 4.8% of global direct health expenditures is attributed to oral diseases with an estimated US$ 323 billion productivity losses arising from oral diseases [1].

Oral health knowledge is considered to be an essential prerequisite for health-related behavior [2, 3]. Good oral hygiene habits are important for maintaining good health and play a vital role in the prevention and in reducing prevalence of oral diseases among adults [4]. Sound oral health knowledge is vital for good overall oral health as an association between increased knowledge and in better healthy practices toward oral health has been established [5]. Adequate oral health knowledge and a positive attitude toward oral health practices have a significant impact on oral health and encourages better oral hygiene behaviors. Oral health education intervention is a significant predictor in reducing the risk of untreated dental caries and in increasing knowledge, attitude, and practices towards oral health [5].

Oral diseases have the potential to cause negative physical, social, and mental consequences across the entire life course of an individual [6]. Oral diseases may impact adolescent or adults at a social level by affecting self-confidence and self-esteem, leading to reduced social interaction, isolation, or even stigmatization and may negatively affect employment opportunities and reduce productivity [1]. In older people, oral diseases may cause pain, impaired chewing and eating ability, or even nutritional deficiencies [7].

A recently conducted meta-analysis indicated that there existed sufficient knowledge of hypothetical observational associations of a bidirectional relationship between oral diseases and noncommunicable diseases [8]. Oral diseases share common modifiable risk factors with noncommunicable diseases, namely, high sugar intake, all forms of tobacco use, and harmful alcohol intake [1], as well as their underlying social and economic determinants. Recognizing the shared risks factors and the inequity in oral health services when compared to noncommunicable diseases, the World Health Organization has prioritized the integration of oral health services with a call for member countries to focus on integrated, population-wide prevention measures, and access to primary oral health care as part of universal health coverage benefit packages [9].

Kuwait is situated in the Persian Gulf with an estimated population of 4.43 million [10] and is one of the richest countries in the world. As a result of the economic transition noncommunicable disease are increasing rapidly, with Kuwait ranked among the top 10 countries with the diabetes prevalence of 25.5% [11]. The main risk factors include the high consumption of fast-foods that have a high salt content, sedentary lifestyle, and inadequate physical activity [12].

Very few studies have been conducted in Kuwait to elicit the knowledge and practices towards oral health amongst adults. In an earlier study, less than two-thirds (62%) of adults in Kuwait followed the recommended toothbrushing frequency of twice daily or more, and majority of adults reported multiple oral health problems, that are mostly preventable through adequate oral hygiene habits and regular preventive dental visits [13]. In a study on oral health status of adult employees in Kuwait, prevalence of caries increased with age among the adults, and females had slightly higher caries prevalence than males [14]. About 19.6% of adult employes had good, 36.1% fair, and 44.4% had poor oral hygiene.

Obtaining primary information of the oral health knowledge and practices is important for implementation of effective prevention programs and for appropriate allocation of dental services for the adults. Also, it will guide the policy makers in evaluating oral health care services. Moreover, this information will strengthen the oral health programs. This study aims to assess the knowledge and practices towards oral health and the association with sociodemographic variables and other factors amongst government employed adults and their clients 18–70 years. Furthermore, this study aims to provide information that could form the basis for targeted oral health promotion programs among adult employes in Kuwait.

## 2. Materials and Methods

### 2.1. Research Design

A cross-sectional study was conducted using an interview administered questionnaire over a period of 5 weeks in 2012.

### 2.2. Setting

The study was conducted at the Ministries Complex and Housing Authority in Kuwait. The sites for the study were conveniently selected. The Ministries Complex and Housing Authority were chosen as they are large government settings in Kuwait and accommodate the main branches of the ministries in the country, where the government employes work. Also, the participants were recruited from these sites, as they are the most feasible locations to conduct the study and to accommodate the participants.

### 2.3. Study Population

A convenience sample of adult employes and clients that provided a service at the offices within the Ministries Complex and Housing Authority were invited to participate. The inclusion criteria for participation were as follows: Adults that worked at the site and clients who attended the Ministries on the date of survey for services. A convenience sample of the clients were recruited. A total of 1302 questionnaires were returned.

### 2.4. Survey Instruments

A customized data collection tool based on previous studies [15, 16] was designed by the principal investigator and dental health team. The tool was translated into Arabic. Data were collected through a customized questionnaire. The questionnaire included 10 demographic questions (age, gender, nationality, employment type, marital status, education level, medical insurance status, and presence of systemic diseases); oral health practices (toothbrush usage: brushing teeth (yes/no), toothpaste use and type, toothbrush replacement, and dental floss use and frequency); nutrition and oral health (fast-food consumption, fruit and vegetables consumption, and eating of sweets); knowledge of dental and oral health (importance of fluoride, reason for gum inflammation, oral cancer, and consumption of healthy-diet and its effect on oral health); risk factors (smoking, water pipe consumption, mouth guard, and bruxism); and overall rating of oral and dental health.

Respondents were provided the following options with respect to nutritional practices (I never eat; consume once a week, twice a week, or more but not daily; and daily). Respondents were provided binary options (yes/no) for two oral health practices questions (toothbrush usage and dental floss use), for four knowledge questions and multiple-choice options for two questions with the correct option being all items listed above. Various sociodemographic variables and selected exposures were included in the multivariate analysis as these are considered important oral health determinants for occurrence of disease.

The team was instructed in overall aspects of administering a questionnaire and oriented to check the recorded responses. The questionnaires were assessed on site for completeness by the team, and the participants were asked to add any missing or incomplete information.

### 2.5. Data Collection

Data were collected by a team of trained dentists over a period of 5 weeks in 2012. The interviews were conducted in a designated room assigned at the Ministries Complex and Housing Authority during the whole survey duration to maintain privacy. The survey was conducted during the office hours of the Ministries Complex and Housing Authority (08:00–15:00). Each participant was informed about the study and its benefits by the dentists as well as via study information sheets circulated to the respective Ministries during the research preparation phase. No personal identification information was obtained from the participants.

### 2.6. Ethical Consideration

The study was approved by the Ethical Research Committee of School Oral Health Program, Kuwait-Forsyth. Target participants were given printed informed consent explaining the aim of the study and assuring them of the anonymity of the collected data. They could refuse participation or withdraw from the study at any time without any consequences. Furthermore, their obligations were made clear about the potential benefits and risks of participating in this study. The collected data were protected by restricted access.

### 2.7. Data Analysis

Data was entered into a Microsoft Excel Spreadsheet that was password protected. Double entry of the data by two independent data capturers were performed. The principal investigator reviewed the completeness and correctness of the data. The data was exported into Statistical software for Data Science Version 17 for analysis.

The sociodemographic of the participants were described using a frequency table. Six questions were posed to assess oral health knowledge of the participants. Each correct answer was scored with a single point. A maximum score of six was attainable for each respondent. A total knowledge score was calculated for each participant and an overall mean and median knowledge score was calculated. The median score (5) was used to classify participants as appropriate knowledge. Bivariate analysis and multivariate logistical analysis was performed to assess the associations between sociodemographic variables and oral health knowledge and practices. Various sociodemographic variables were included in the multivariate analysis (logistic regression) as these are considered important oral health determinants for occurrence of disease. Only variables with *p* < 0.2 were inserted into the multivariable logistical regression model.

## 3. Results

### 3.1. General Population Characteristics

Table 1 presents a description of the general characteristics of the study population. The mean age of the study population was 32 years (SD: 12) with a median of 27 years (IQR: 24−42). Sixty seven percent (*n* = 879) of the respondents were below 40 years of age, 67% (*n* = 851) were males, and 67% (*n* = 866) were Kuwaitis. Seventy seven percent (*n* = 1004) of the respondents were healthy individuals, whilst 9% had hypertension (5%), diabetes (3%), and cardiac diseases (1%). Forty seven percent (*n* = 599) of the respondents visited the dentist within the last 6 months. Seventy percent (*n* = 899) visited the dentist for a specific dental health problem. Twenty five percent (*n* = 324) of the study population smoked cigarets, whilst 15% (*n* = 188) used water pipes. Sixty nine percent (*n* = 883) of the study population consumed fast-foods, with 50% consuming fast-foods more than once a week. Sixty five percent (*n* = 833) and 63% (*n* = 814), respectively, indicated that they consumed fruits and vegetables consistently. Ninety eight percent of the population (*n* = 1258) consumed sugary sweets more than once a week.

### 3.2. Oral Health Practices

Table 2 highlights the oral health practices of the respondents. Most respondents (93%) brushed their teeth. Eighty nine percent (*n* = 1115) brushed their teeth once or more during the day with 40% (*n* = 499) brushing twice a day and 20% (*n* = 254) brushing more than twice a day. Ninety four percent (*n* = 1168) utilized toothpaste. Sixty three percent (*n* = 803) changed their toothbrushes every 6 months or less. Flossing was less common amongst the respondents with only 28% (*n* = 340) responding affirmatively to the practice. Of those, using floss only 23% used it consistently (Table 2).

### 3.3. Oral Health Knowledge

**Figure 1:**
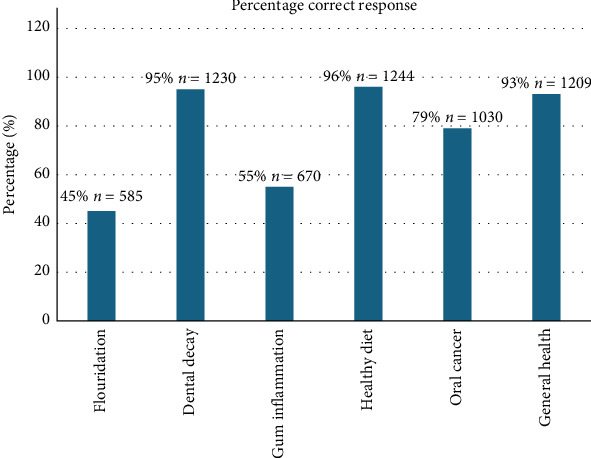
Proportion of respondents with correct responses to knowledge questions.

The maximum attainable knowledge score for an individual respondent was 6. The overall mean knowledge score for all participants was 4.59 (SD: 1.05) with a median of 5 (IQR: 4–5). Respondents obtained a lower score for the importance of fluoridation and reason for gum inflammation (Figure 1).

### 3.4. Association Between Sociodemographic Factors and Participants Oral Health Knowledge and Practices

#### 3.4.1. Oral Health Knowledge

Table 3 provides a summary of the frequency distribution as well as bivariate and multivariate analysis of the sociodemographic factors and oral health knowledge. The participants were classified as knowledge above median (5) versus below median. The value of 5 was selected as it is in the opinion of the authors that a score of 80% and above was considered good oral health knowledge.

On bivariate analysis two variables: respondents that were Kuwaiti (UOR: 1.58; 95% CI: 1.25–2.01) and those having a post school education (UOR: 2.78; 95% CI: 1.83–4.2) showed significant increased odds of above median knowledge. Male participants (UOR: 0.4; 95% CI: 0.31–0.52) and participants who were smokers (UOR: 0.41; 95% CI: 0.32–0.54) were significantly less likely to have an above median knowledge score. After multivariate analysis using only significant factors in the model having a post school education was the only variable significantly associated with an above median knowledge (AOR: 2.34; 95% CI: 1.50–3.66). Male participants (AOR: 0.51; 95% CI: 0.38–0.69) and participants who were smokers (AOR: 0.56; 95% CI: 0.43–0.77) were significantly less likely to have an above median knowledge score. In addition, respondents that ate sugary sweets at least once a month (AOR: 0.66; 95% CI: 0.44–0.98) and those that consumed sugary sweets daily (AOR: 0.68; 95% CI: 0.46–0.99) had a significantly lower median knowledge score than those did not consume sugary sweets (Table 3).

#### 3.4.2. Associations With Oral Hygiene Practices

Table 4 summarizes the association between sociodemographic factors and oral hygiene practices. On bivariate analysis, participants with a post school education (UOR: 4.68; 95% CI: 2.59–8.45), having a dental visit in the last 6 months or less (UOR: 3.94; 95% CI: 1.34–6.64) or 6 months or more since last dental visits (UOR: 2.07; 95% CI: 1.10–3.89) respondents who consumed sugary sweets daily (UOR: 2.87; 95% CI: 1.49–5.51) as well as participants with above median knowledge scores (UOR: 1.98; 95% CI: 1.25–3.15) were significantly more likely to brush their teeth. On the contrary, male participants (UOR: 0.21; 95% CI: 0.0005–0.12) and smokers (UOR: 0.31; 95% CI: 0.19–0.49) were significantly less likely to brush their teeth.

After multivariate analysis, post school education (AOR: 2.67; 95% CI: 1.39–5.16), 6 months or less since last dental visit (AOR: 3.21; 95% CI: 1.81–5.69), 6 months or more since last dental visits (AOR: 2.67; 95% CI: 1.39–5.16), and participants who consumed sugary sweets daily (AOR: 2.38; 95% CI: 1.16–4.30) remained significantly associated with brushing teeth. Male participants (AOR: 0.41; 95% CI: 0.0005–0.31) and smokers (AOR: 0.59; 95% CI: 0.36–0.96) were significantly less likely to brush their teeth (Table 4).

##### 3.4.2.1. Dental Flossing

Table 5 summarizes the association between sociodemographic factors and dental flossing. After bivariate analysis, participants from the following age groups 30–39 (UOR: 1.55; 95% CI: 1.13–2.12), 40–49 (UOR: 1.97; 95% CI: 1.39–2.79), 50–59 (UOR: 1.6; 95% CI: 1.03–2.50), Kuwaiti nationals (UOR: 3.05; 95% CI: 2.22–4.24), respondents with post high school education (UOR: 2.42; 95% CI: 1.39–4.21), those who had the last dental visit 6 months ago (UOR: 2.95; 95% CI: 1.96–4.42) or the last dental visit 6 months or more (UOR: 1.97; 95% CI: 1.29–3.02), and those who consumed vegetables daily (UOR: 8.12; 95% CI: 1.09–61.06) as well as respondents with above median knowledge scores (UOR: 1.47; 95% CI: 1.12–1.91) were significantly more likely to floss their teeth. On the contrary male participants (UOR: 0.61; 95% CI: 0.47–0.80), participants with medical insurance (UOR: 0.44; 95% CI: 0.33–0.59), smokers (UOR: 0.63; 95% CI: 0.45–0.87), and those that used a water pipe (UOR: 0.62; 95% CI 0.40–0.95) were significantly less likely to floss their teeth.

After multivariate analysis, respondents that were between 30 and 39 years (AOR: 1.65; 95% CI: 1.17–2.33), 40–49 years (UOR: 2.02; 95% CI: 1.35–3.04), 50–59 years (AOR: 1.85; 95% CI: 1.08–3.15), Kuwaiti nationals (AOR: 2.97; 95% CI: 1.84–4.80), and with post high school education (UOR: 2.61; 95% CI: 1.66–4.11) last dental visit 6 months or less (AOR: 2.62; 95% CI: 1.66–4.12) or 6 months or more since last dental visits (AOR: 1.78; 95% CI: 1.11–2.87) were significantly more likely to floss their teeth. Only participants who used a water pipe (AOR: 0.57; 95% CI 0.36–0.92) were significantly less likely to floss their teeth (Table 5).

## 4. Discussion

This survey is amongst the first that have been conducted on an adult population in Kuwait. Although the sample was conveniently selected and included easy to access individuals, the study findings shed important information towards developing tailored strategies for different population age groups to attain maximum benefit.

The surveyed population displayed several high-risk behaviors such as smoking (25%), use of water pipes (15%), consumption of fast-food (69%), and consumption of sugary sweets (98%), characteristics that predispose an individual to poor overall dental health. There is increasing evidence that the consumption of unhealthy food and beverages is a major contributor to oral diseases [17]. Research has consistently shown the link between free sugars and dental caries. Data show a dose–response relationship between sugar consumption and dental caries; the higher the sugar intake, the higher the caries experience and severity of the disease [18]. Sugar acts as a substrate for oral bacteria that produce demineralizing acids [19], whilst the consumption of acidic foods and beverages leads to dental erosion, which can lead to cavities or (dental) hypersensitivity [20]. Furthermore, it has been shown that the consumption of saturated fatty acids may promote the growth of certain proteolytic bacteria leading to periodontal disease [21]. Kuwait as a high-income country is experiencing a nutritional transition from home-cooked foods to processed sugary snacks and drinks and fast-foods. Not only does this have negative implications for the adults, but the concern is that the children of these adults will be at greater risk of poor oral health because of excessive sugar and salt intake.

Oral health knowledge is important for good overall oral health as an association between increased knowledge and better oral health has been demonstrated [5]. In our study, respondents had a good level of knowledge with deficiencies in two areas, namely, importance of fluoridation and reason for gum inflammation. In a previous study in Saudi Arabia, oral health knowledge was low among the university students [22].

Overall, participants knowledge with respect to oral health was very good with a mean of 4.59 and a median of 5.0. Although, we used different survey tools, the above knowledge scores are similar to a study conducted in the school teachers in India [23] and in a study among school teachers in Iran [24]. Respondents obtained a lower score for the importance of fluoridation and reason for gum inflammation. Higher level of education was associated with better oral health knowledge scores (AOR: 2.34; 95% CI: 1.50–3.66). A study conducted at the University of Valencia Dental Clinic in Spain showed that as subjects' educational level increased, so did their level of oral health knowledge [25].

Females were more likely than males to have an above median knowledge score. Several previous studies have shown that females have better oral health knowledge than males [4, 16, 22, 26]. Previous studies have attributed the better oral health knowledge amongst females due to their intrinsic desire for a good body image. As a result females tend to care more about their health and are more likely to follow a diet to control their weight and oral health, visit the dentist more frequently, and this could help them acquire more knowledge about oral health [4].

Participants who were smokers (AOR: 0.56; 95% CI: 0.43–0.77) were significantly less likely to have an above median knowledge score. These findings were consistent with a study in Indonesia amongst adolescent students [27] and in Nigeria [28].

Brushing of teeth and usage of toothpaste was performed by most of the respondents. Two main areas of concern from our findings were that smokers and males were significantly less likely to brush their teeth. A population-based survey amongst 4485 adults aged 18+ years, in Chongqing city, China, showed that more than half of the adults who rarely or never brushed their teeth reported themselves as smoking when the survey was conducted [29]. Epidemiological studies have shown an association between smoking and periodontitis and have identified smoking as an important risk factor for oral cancer [29].

Dental flossing has been shown to enhance the benefits of toothbrushing by reducing interdental plaque build-up and halting its progression, while improving gingival health in adults [ 30]. In the current study, dental flossing was practiced by 27% of the respondents at least once a month with only a minority flossing daily. The American National Health and Nutrition Examination Survey 2011–2014 indicated that 31.6% of adults flossed daily, with respondents more than 30 years of age more likely to floss when compared to younger than 30 years [31]. Age, level of education, and recent last dental were significantly associated with flossing of teeth in the current study. These findings are consistent with survey to determine oral hygiene behaviors among Australian adults in the National Study of Adult Oral Health (NSAOH) 2017–18 that showed better socioeconomic status and regular and recent dental health visits were associated with dental flossing [32].

Although scientific integrity and ethical standards were maintained in conducting the survey, the study has several limitations. The study was conducted on employed adults within the Ministerial Complex or visitors to the complex. This is a biased study sample as it does not represent the population profile of Kuwait, and therefore, the results may not be generalizable. Also, the research was performed based on self-reported data. Thus, participants may have made errors in interpreting the questions. Moreover, another limitation was this survey was based on a cross-sectional study design to test associations, which prevents to infer causality.

Despite the limitations, the study provides baseline information that can be utilized to plan oral health promotion practices for this population group. The lessons learnt from this study provide a platform for expanding the survey to the general population to expand preventive health promotion interventions.

## 5. Conclusions

Employed adults within the Ministerial Complex in Kuwait demonstrate good oral health knowledge, however, daily dental flossing is inadequately practiced. Nutritional practices such as fast-food consumption and sugary sweet consumption predispose this population to adverse oral health outcomes. Males and smokers have poorer knowledge and inadequate oral hygiene practices. In order to improve the overall oral health status, it is important that a population-based approach utilizing policy imperatives, screening programs for oral health, school-based health, oral health promotion, and screening. Early initiation of treatment and social marketing programs targeting risk reduction behavior is implemented. This study highlights the need for educational interventions focusing on comprehensive oral hygiene practices among adults. Further efforts are essential to increase awareness in workplaces to help encourage adult employes in Kuwait in developing healthy oral health practices. Future periodic studies should be planned to assess trends in oral health among adults in Kuwait.

## Figures and Tables

**Table 1:** General characteristics of study population.

Variable	Categories	Total population (number) = 1275	Total population (%)
Age	18–29	448	34
	30–39	431	33
	40–49	257	20
	50–59	132	10
	60–69	30	2
	>70	3	1

Gender	Female	425	33
	Male	851	67

Nationality	Non-Kuwaiti	430	33
	Kuwaiti	866	67

Marital status	Single	895	70
	Married	380	30

Education	Intermediate and lower	106	8
	High school	291	23
	Post school	885	69

Medical insurance	No	779	60
	Yes	470	38

Parents with children	No	438	34
	Yes	842	66

Do you work in the Ministry Complex or Housing Authority?	No	182	14
	Yes	1068	85

Chronic diseases	1. Others	116	9
	2. Blood disease	9	1
	3. Hypertension	60	5
	4. Diabetic	38	3
	5. Cardiac diseases	13	1
	6. Healthy–normal	1004	77

Last dental visit	I do not remember	238	19
	Six months or less	445	35
	More than 6 months	600	47

Smoking	No	975	76
	Yes	307	24

Water pipe	No	1112	86
	Yes	170	14

Fast-food consumption	No	399	31
	Yes	884	69

Frequency of fast-food consumption	I never eat fast-food	78	8
	Maybe once a month	442	43
	Once a week	188	18
	Twice a week	327	31

Fresh vegetables consumption	I never eat fruit	20	2
	Maybe once a month	91	7
	Minimum once a week	344	27
	Daily	834	65

Do you eat fresh fruits?	I never eat fruit	14	1
	Maybe once a month	85	7
	Minimum once a week	376	29
	Daily	816	63

Sugary sweets	I never eat	197	15
	Maybe once a month	325	25
	Minimum once a week	327	25
	Daily	440	34

**Table 2:** Oral health practices of study population.

Variable	Categories	Total population (number) = 1277	Total population (%)
Brushing teeth	No	89	7
	Yes	1199	93

Frequency of brushing teeth	Not every day	149	12
	Once a day	362	29
	Twice a day	499	40
	More than twice a day	254	20

What do you use with your toothbrush?	Others	29	2
	Tooth whitening powder	24	2
	Sodium bicarbonate	21	2
	Toothpaste	1168	94

How often do you change your toothbrush?	I can not remember	56	4
	When the brush is spoilt	236	19
	A year or more	172	14
	Six months or less	802	63

Do you use dental floss?	No	937	72
	Yes	340	28

How many times do you floss, if your previous answer was yes?	I can not remember	426	33
	I use it sometimes	199	24
	Once or more per month	57	7
	Once or more per day	132	16

**Table 3:** Association between sociodemographic variables and oral health knowledge.

Variable	Categories	Above median knowledge (5) *N* (%)	Below median knowledge *n* (%)	Unadjusted OR	95% CI	*p* value	Adjusted OR	95% CI	*p* value
Age	18–29	247 (19)	201 (15)	Reference					
	30–39	251 (19)	180 (14)	1.14	0.87–1.48	0.35	—	—	—
	40–49	148 (11)	109 (8)	1.11	0.81–1.5	0.52	—	—	—
	50–59	83 (6)	49 (4)	1.34	0.92–2.06	0.12	—	—	—
	>60 years	13 (1)	20 (2)	0.53	0.26–1.09	0.09	—	—	—

Gender	Female	303 (24)	122 (10)	Reference					
	Male	423 (33)	428 (34)	0.4	0.31–0.52	<0.001*⁣*^*∗∗*^	0.51	0.38–0.69	C;0.001*⁣*^*∗∗*^

Nationality	Non-Kuwaiti	213 (16)	217 (17)	Reference					
	Kuwaiti	527 (41)	339 (26)	1.58	1.25–2.01	<0.001*⁣*^*∗∗*^	1.04	0.79–1.39	0.74

Marital status	Single	515 (40)	380 (30)	Reference					
	Married	208 (16)	172 (14)	1.05	0.94–1.17	0.36	—	—	—

Education	Intermediate and lower	40 (3)	66 (5)	Reference					
	High school	138 (11)	153 (12)	1.49	0.95–2.34	0.09	1.42	0.87–2.29	0.16
	Post school	555 (43)	330 (26)	2.78	1.83–4.2	<0.001*⁣*^*∗∗*^	2.34	1.50–3.66	<0.001*⁣*^*∗∗*^

Medical insurance	No	464 (37)	315 (25)	Reference					
	Yes	245 (20)	225 (18)	1.14	1.03–1.27	0.01*⁣*^*∗*^	0.98	0.69–1.38	0.92

Parents with children	No	239 (19)	199 (16)	Reference					
	Yes	492 (38)	350 (27)	1.17	0.92–1.48	0.19	—	—	—

Do you work in the Ministry Complex or Housing Authority?	No	121 (10)	110 (9)	Reference					
	Yes	600 (58)	429 (42)	1.25	0.90–1.74	0.1	—	—	—

Chronic disease	No	605 (83)	453 (35)	Reference					
	Yes	127 (10)	97 (8)	1.01	0.89–1.15	0.88	—	—	—

Last dental visit	I do not remember	125 (10)	113 (9)	Reference					
	Six months or less	360 (28)	240 (19)	1.18	0.86–1.62	0.3	—	—	—
	More than 6 months	252 (20)	193 (15)	1.36	1–1.84	0.05	—	—	—

Smoking	No	608 (47)	367 (29)	Reference					
	Yes	124 (10)	183 (14)	0.41	0.32–0.54	<0.001*⁣*^*∗∗*^	0.56	0.43–0.77	<0.001*⁣*^*∗∗*^

Water pipe	No	648 (50)	464 (36)	Reference					
	Yes	85 (7)	85 (7)	0.72	0.51–1.00	0.04	1.06	0.76–1.52	0.34

Fast-food consumption	No	220 (17)	179 (14)	Reference					
	Yes	512 (40)	372 (29)	1.12	0.87–1.43	0.35	—	—	—

Frequency of fast-food consumption	I never eat fast-food	38 (4)	40 (4)	Reference					
	Maybe once a month	259 (25)	183 (18)	1.49	0.92–2.41	0.11	—	—	—
	Once a week	101 (10)	87 (8)	1.22	0.79–2.07	0.46	—	—	—
	Twice a week	180 (17)	147 (14)	1.29	0.79–2.11	0.31	—	—	—

Fresh vegetables consumption	I never eat fruit	9 (1)	11 (1)	Reference					
	May be once a month	49 (4)	42 (3)	1.38	0.49–3.83	0.55	—	—	—
	Minimum once a week	186 (14)	158 (12)	1.47	0.56–3.85	0.44	—	—	—
	Daily	495 (38)	339 (26)	2.07	0.79–5.42	0.14	—	—	—

Do you eat fresh fruits?	I never eat fruit	8 (1)	6 (1)	Reference					
	Maybe once a month	47 (4)	38 (3)	0.88	0.26–2.98	0.84	—	—	—
	Minimum once a week	214 (17)	162 (13)	0.92	0.29–2.93	0.89	—	—	—
	Daily	469 (36)	347 (27)	0.76	0.24–2.4	0.64	—	—	—

Sugary sweets	I never eat	221 (9)	76 (6)	Reference					
	Maybe once a month	186 (14)	139 (11)	0.84	0.58–1.22	0.36	0.66	0.44–0.98	0.04*⁣*^*∗*^
	Minimum once a week	174 (14)	153 (12)	0.69	0.48–1.0	0.07	0.47	0.47–1.04	0.08
	Daily	256 (20)	184 (14)	0.85	0.60–1.2	0.35	0.68	0.46–0.99	0.05*⁣*^*∗*^

*Note*: No input in the model.

⁣^*∗*^*p* < 0.05, ⁣^*∗∗*^*p* < 0.001.

**Table 4:** Association between sociodemographic factors and brushing of teeth.

Variable	Categories	Not brushing teeth	Brushing teeth	Unadjusted OR	95% CI	*p* value	Adjusted OR	95% CI	*p* value
Age	18–29	40 (3%)	403 (31%)	Reference					
	30–39	24 (2%)	403 (31%)	1.67	0.98–2.82	0.06	—	—	—
	40–49	15 (1%)	239 (19%)	1.58	0.86–2.92	0.14	—	—	—
	50–59	10 (1%)	122 (9%)	1.21	0.58–2.49	0.6	—	—	—
	>60 years	0	32 (2%)	—	—	—	—	—	—

Gender	Female	1 (0.08%)	423 (33%)	Reference					
	Male	85 (7%)	754 (60%)	0.21	0.0005–0.12	<0.001*⁣*^*∗∗*^	0.41	0.005–0.31	0.001*⁣*^*∗∗*^

Nationality	Non-Kuwaiti	52 (4%)	372 (29%)	Reference					
	Kuwaiti	37 (3%)	822 (64%)	3.11	1.96–4.95	<0.001*⁣*^*∗∗*^	1.57	0.94–2.62	0.08

Marital status	Single	57 (4%)	831 (66%)	Reference					
	Married	31 (2%)	343 (27%)	0.76	0.47–1.24	0.36	—	—	—

Education	Intermediate and lower	19 (2%)	85 (7%)	Reference					
	High school	28 (2%)	259 (20%)	2.07	1.10–3.89	0.02	1.97	0.98–3.97	0.06
	Post school	40 (3%)	838 (65%)	4.68	2.59–8.45	<0.001*⁣*^*∗∗*^	2.67	1.39–5.16	0.003*⁣*^*∗*^

Medical insurance	No	45 (4%)	725 (59%)	Reference					
	Yes	38 (3%)	428 (35%)	0.7	0.44–1.13	0.12	—	—	—

Parents with children	No	37 (3%)	395 (31%)	Reference					
	Yes	51 (4%)	784 (62%)	1.44	0.91–2.28	0.1	—	—	—

Do you work in the Ministry Complex or Housing Authority?	No	14 (1%)	213 (17%)	Reference					
	Yes	71 (6%)	950 (76%)	0.88	0.44–1.61	0.67	—	—	—

Chronic disease	No	69 (5%)	977 (77%)	Reference					
	Yes	19 (2%)	203 (16%)	0.75	0.44–1.36	0.29	—	—	—

Last dental visit	I do not remember	37 (3%)	198 (16%)	Reference					
	Six months or less	24 (2%)	414 (33%)	3.94	12.34–6.64	<0.001*⁣*^*∗∗*^	3.21	1.81–5.69	C;0.001*⁣*^*∗∗*^
	More than 6 months	27 (2%)	570 (45%)	3.22	1.88–5.53	<0.001*⁣*^*∗∗*^	2.68	1.49–4.82	0.001*⁣*^*∗∗*^

Smoking	No	46 (4%)	922 (73%)	Reference					
	Yes	42 (3%)	259 (21%)	0.31	0.19–0.49	<0.001*⁣*^*∗∗*^	0.59	0.36–0.96	0.04

Water pipe	No	70 (6%)	1030 (81%)	Reference					
	Yes	15 (1%)	154 (12%)	0.7	0.38–1.35	0.22	—	—	—

Fast-food consumption	No	24 (2%)	368 (29%)	Reference					
	Yes	64 (5%)	814 (64%)	0.83	0.49–1.37	0.45	—	—	—

Frequency of fast-food consumption	I never eat fast-food	5 (0.5%)	73 (7%)	Reference					
	Maybe once a month	32 (3%)	406 (40%)	0.87	0.33–2.30	0.78	—	—	—
	Once a week	5 (0.5%)	178 (17%)	2.44	0.68–8.67	0.17	—	—	—
	Twice a week	32 (3%)	294 (29%)	0.63	0.24–1.67	0.35	—	—	—

Fresh vegetables consumption	I never eat fruit	3 (0.24)	16 (1%)	—	—	—	—	—	—
	Maybe once a month	49 (4%)	42 (3%)	1.5	0.37–6.06	0.57	—	—	—
	Minimum once a week	186 (14%)	158 (12%)	2.59	0.70–9.54	0.44	—	—	—
	Daily	495 (38%)	339 (26%)	2.79	0.79–9.88	0.11	—	—	—

Do you eat fresh fruits?	I never eat fruit	1 (0.08%)	13 (1%)	Reference					
	Maybe once a month	8 (1%)	76 (6%)	0.73	0.84–6.33	0.78	—	—	—
	Minimum once a week	26 (2%)	345 (27%)	1.02	0.13–8.11	0.99	—	—	—
	Daily	53 (4%)	756 (59%)	1.09	0.15–8.55	0.93	—	—	—

Sugary sweets	I never eat	21 (2%)	171 (13%)	Reference			—	—	—
	Maybe once a month	21 (2%)	301 (24%)	1.76	0.93–3.32	0.08	1.22	0.61–2.50	—
	Minimum once a week	29 (2%)	295 (23%)	1.24	0.69–2.26	0.46	1.51	0.79–2.89	0.22
	Daily	18 (1%)	420 (33%)	2.87	1.49–5.51	0.002	2.38	1.16–4.90	0.002*⁣*^*∗*^

Oral dental knowledge	Below median	52 (4%)	498 (38%)	Reference					
	Above median	37 (3%)	701 (54%)	1.98	1.25–3.15	0.002	1.29	0.79–2.13	0.3

*Note*: No input in the model.

*⁣* ^*∗*^ *p* < 0.05, *⁣*^*∗∗*^*p* < 0.001.

**Table 5:** Association between sociodemographic factors and dental flossing.

Variable	Categories	Dental flossing (No) *N* (%)	Dental flossing (Yes) *n* (%)	Unadjusted OR	95% CI	*p* value	Adjusted OR	95% CI	*p* value
Age	18–29	350 (27)	90 (7)	Reference					
	30–39	304 (23)	121 (9)	1.55	1.13–2.12	0.006	1.65	1.17–2.33	0.004
	40–49	166 (13)	84 (6)	1.97	1.39–2.79	<0.0001*⁣*^*∗∗*^	2.02	1.35–3.04	0.001
	50–59	92 (7)	38 (3)	1.6	1.03–2.50	0.03	1.85	1.08–3.15	0.03
	>60 years	25 (2)	7 (1)	1.09	0.45–2.59	0.85	1.67	0.64–4.39	0.3

Gender	Female	281 (22)	138 (11)	Reference					
	Male	641 (49)	193 (15)	0.61	0.47–0.80	0.0002	1.01	0.72–1.41	0.96

Nationality	Non-Kuwaiti	362 (28)	58 (4)	Reference					
	Kuwaiti	572 (44)	280 (22)	3.05	2.22–4.24	<0.0001*⁣*^*∗∗*^	2.97	1.84–4.80	<0.000*⁣*^*∗∗*^

Marital status	Single	639 (49)	238 (18)	Reference					
	Married	277 (21)	98 (8)	0.95	0.71–1.26	0.71	—	—	—

Education	Intermediate and lower	87 (7)	16 (1)	Reference					
	High school	229 (18)	53 (4)	1.26	0.68–2.32	0.46	1.25	0.61–2.52	0.02
	Post school	604 (46)	269 (21)	2.42	1.39–4.21	0.002	2.61	1.66–4.11	<0.000*⁣*^*∗∗*^

Medical insurance	No	518 (40)	248 (19)	Reference					
	Yes	380 (29)	80 (6)	0.44	0.33–0.59	<0.0001*⁣*^*∗∗*^	0.83	0.55–1.25	0.37

Parents with children	No	320 (25)	110 (8)	Reference					
	Yes	604 (43)	222 (17)	1.07	0.81–1.41	0.62	—	—	—

Do you work in the Ministry Complex or Housing Authority?	No	160 (12)	68 (5)	Reference					
	Yes	744 (57)	264 (20)	0.84	0.60–1.16	0.26	—	—	—

Chronic disease	No	767 (59)	272 (21)	Reference					
	Yes	156 (12)	62 (5)	1.12	0.80–1.57	0.49	—	—	—

Last dental visit	I do not remember	199 (15)	33 (3)	Reference	—	—	—	—	—
	Six months or less	397 (31)	194 (15)	2.95	1.96–4.42	<0.0001*⁣*^*∗∗*^	2.62	1.66–4.12	0.02
	More than 6 months	330 (25)	108 (5)	1.97	1.29–3.02	0.002	1.78	1.11–2.87	0.05

Smoking	No	679 (52)	275 (21)	Reference					
	Yes	242 (19)	62 (5)	0.63	0.45–0.87	0.003	0.9	0.61–1.32	0.58

Water pipe	No	789 (61)	304 (23)	Reference					
	Yes	133 (10)	32 (2)	0.62	0.40–0.95	0.02	0.57	0.36–0.92	0.02*⁣*^*∗*^

Fast-food consumption	No	284 (22)	106 (8)	—	—	—	—	—	—
	Yes	640 (49)	233 (18)	0.98	0.74–1.29	0.86	—	—	—

Frequency of fast-food consumption	I never eat fast-food	62 (5)	15 (1)	Reference					
	Maybe once a month	311 (24)	122 (9)	1.62	0.88–2.96	0.12	—	—	—
	Once a week	134 (10)	51 (4)	1.57	0.82–3.01	0.17	—	—	—
	Twice a week	247 (19)	74 (6)	1.24	0.67–2.30	0.5	—	—	—

Fresh vegetables consumption	I never eat fruit	3 (0.24)	16 (1)	Reference					
	Maybe once a month	49 (4)	42 (3)	3.85	0.48–31.01	0.21	3.66	0.44–30.51	0.23
	Minimum once a week	186 (14)	158 (12)	5.53	0.73–42	0.1	5.14	0.66–40.16	0.12
	Daily	495 (38)	339 (26)	8.12	1.09–61.06	0.04	7.53	0.97–58.26	0.05

Do you eat fresh fruits?	I never eat fruit	10 (1)	4 (0.3)	Reference					
	Maybe once a month	67 (5)	16 (1)	0.6	0.17–2.15	0.43	—	—	—
	Minimum once a week	272 (21)	97 (7)	0.89	0.27–2.91	0.85	—	—	—
	Daily	579 (45)	223 (17)	0.97	0.30–3.10	0.95	—	—	—

Sugary sweets	I never eat	144 (11)	43 (3)	Reference					
	Maybe once a month	238 (18)	83 (6)	1.17	0.77–1.78	0.47	—	—	—
	Minimum once a week	237 (18)	84 (6)	1.19	0.78–1.81	0.43	—	—	—
	Daily	309 (24)	127 (10)	1.38	0.92–2.05	0.12	—	—	—

Oral dental knowledge	Below median	423 (33%)	122 (9%)	Reference					
	Above median	514 (40%)	218 (17%)	1.47	1.12–1.91	0.003	1.13	0.84–1.53	0.41

*Note*: No input in the model.

*⁣* ^*∗*^ *p* < 0.05, *⁣*^*∗∗*^*p* < 0.001.

## Data Availability

The data are available on request from the first author.
